# Meta-Analysis of Repeat Hepatectomy versus Radiofrequency Ablation for Recurrence of Hepatocellular Carcinoma

**DOI:** 10.3390/cancers14215398

**Published:** 2022-11-02

**Authors:** Nikolaos Machairas, Dimitrios Papaconstantinou, Panagiotis Dorovinis, Diamantis I. Tsilimigras, Myrto D. Keramida, Stylianos Kykalos, Dimitrios Schizas, Timothy M. Pawlik

**Affiliations:** 1 2nd Department of Propaedeutic Surgery, National and Kapodistrian University of Athens, 11527 Athens, Greece; 2 3rd Department of Surgery, National and Kapodistrian University of Athens, 12462 Chaidari, Greece; 3 Department of Surgery, Division of Surgical Oncology, The Ohio State University Wexner Medical Center and James Comprehensive Cancer Center, Columbus, OH 43210, USA; 4 1st Department of Surgery, National and Kapodistrian University of Athens, 11527 Athens, Greece

**Keywords:** hepatocellular carcinoma, hepatectomy, recurrence, ablation, repeat hepatectomy, survival

## Abstract

**Simple Summary:**

Development of intrahepatic recurrence of HCC is common even following index curative-intent hepatectomy. Multiple studies have demonstrated that repeat hepatectomy (RH) or radiofrequency ablation (RFA) may be performed in patients with recurrent disease. This systematic review and meta-analysis aimed to compare short- and long-term outcomes of patients undergoing RHR versus RFA for recurrent HCC.

**Abstract:**

Hepatocellular carcinoma (HCC) is the most common primary hepatic malignancy and a leading cause of cancer-related death in both the developed and developing world. Recurrent HCC (rHCC) develops in a significant proportion of patients even following curative-intent resection. In the absence of a structured treatment algorithm, a number of treatment options including repeat hepatectomy (RH) and radiofrequency ablation (RFA) have been utilized in select patients with rHCC. The aim of this systematic review and meta-analysis was to compare short- and long-term outcomes of patients undergoing RHR versus RFA for rHCC. Four electronic databases were screened until September 2022. A total of 17 studies were included in the meta-analysis. Overall and disease-free survival were comparable among the two groups. Patients undergoing RH were less likely to develop a second recurrence (RR 0.89, 95% C.I. 0.81 to 0.98, *p* = 0.02). Overall and major morbidity were significantly increased in the RH group (RR 3.01, 95% C.I. 1.98 to 4.56, *p* < 0.001 and RR 3.65, 95% C.I. 2.07 to 6.43, *p* < 0.001, respectively), while mortality was similar between RFA and RH. The data demonstrated that RFA is a safe and efficient alternative to RH for selected patients with rHCC. Nevertheless, despite higher morbidity associated with RH, repeat resection remains the preferred treatment option whenever feasible, as it allows for better local disease control.

## 1. Introduction

Hepatocellular carcinoma (HCC) is the most frequently diagnosed primary hepatic malignancy, which is currently the sixth most common cancer type and third leading cause of cancer-related death around the world [1,2]. Chronic hepatitis B and C virus (HBV, HCV) infections, alcoholic liver disease (ALD), non-alcoholic fatty liver disease (NAFLD), as well as non-alcoholic steatohepatitis (NASH) are associated with HCC risk [3,4,5]. The Barcelona Clinic Liver Cancer (BCLC) staging classification system provides a tool for stratification and treatment allocation of newly diagnosed patients with HCC in the setting of cirrhosis [6,7]. Radiofrequency ablation (RFA) is indicated for highly selected patients with very early (0) and early stage (A) disease (single or up to 3 nodules ≤ 3 cm, preserved liver function and good performance status) that are not candidates for transplantation or resection [8]. In addition to liver transplantation, which may be the optimal treatment option for patients with HCC who fulfill criteria, surgical resection remains the mainstay for selected patients (BCLC 0 and A) as the only potentially curative option yielding the best chance at long-term overall survival [8,9,10,11,12,13].

Recurrence of HCC is common even among patients with ablated or margin negative (R0) resected lesions; the incidence of intrahepatic recurrence has been reported to be approximately 50–70% within 5-years [14]. Surgical treatment options for selected patients with recurrent HCC (rHCC) may include salvage liver transplantation, repeat hepatectomy, and radiofrequency ablation (RFA), although there is currently no definitive classification systems or algorithms for the treatment of these patients [8,15,16,17]. Repeat hepatic resection (RHR) remains the treatment of choice for well-selected patients presenting with intrahepatic recurrence who have good performance status, an adequate functional liver remnant (FLR) and technically resectable disease [18]. In contrast, among patients with poor performance status, progressive liver disease, small residual liver volume and possible technical difficulties following re-resection, RFA has been demonstrated to be a safe alternative option. As with primary HCC, RFA has been associated with decreased intraoperative complications, reduced blood loss and shorter length of hospital stay [19]. 

In the absence of a standardized algorithm for the treatment of rHCC, RHR remains the optimal option, though emerging data suggest that RFA represents a safe and efficient alternative. To that end, the objective of the current systematic review and meta-analysis was to compare short- and long-term outcomes of patients undergoing RHR versus RFA for rHCC. 

## 2. Materials and Methods

### 2.1. Literature Search

The meta-analysis was designed according to the Preferred Reporting Items for Systematic Reviews and Meta-Analyses (PRISMA) guidelines and based on predetermined eligibility criteria [20]. The systematic review was preregistered with the International Prospective Register of Systematic Reviews (PROSPERO, reg. no. CRD420222357301). 

A systematic search of the PubMed, Scopus, Web of Science and Cochrane databases for articles published up to September 2022 was conducted by three independent authors (N.M., P.D. and M.D.K.) with any ensuing disagreements or discrepancies resolved by consensus among all authors. The reference lists of all potentially eligible articles were manually checked for additional relevant studies. The systematic search protocol included the keywords: “radiofrequency ablation”, “ablation”, “liver resection”, “hepatic resection”, “hepatectomy”, “repeat liver resection”, “redo liver resection“ and ”recurrent hepatocellular carcinoma”. Publications that fulfilled or were considered to fulfill the eligibility criteria were retrieved in full text. 

### 2.2. Inclusion and Exclusion Criteria

Clinical studies reporting peri-procedural and/or long-term oncologic outcomes of adult patients with recurrent HCC undergoing RHR or RFA with a curative intent were considered eligible. A set of predetermined exclusion criteria was utilized to minimized clinical heterogeneity among the included studies and to guide the study selection process: (1) Non-clinical studies and case reports, (2) studies in which primary HCC was treated by modalities other than liver resection, (3) non-comparative studies or studies not reporting comparative outcomes between RHR and RFA patient populations, (4) studies not evaluating any periprocedural or survival outcomes, (5) studies reporting oncologic survival outcomes studies with overlapping patient populations, (6) non-English studies.

### 2.3. Data Extraction and Outcomes of Interest

After full-text review of all studies deemed eligible for inclusion in the quantitative analysis, data were extracted and entered into standardized excel spreadsheets (Microsoft, Redmond, DC, USA) by two authors (P.D and M.D.K), while a third author (D.P.) reviewed the data for any discrepancies. Primary outcomes of interest were the Hazard Ratios (HR) for Overall Survival (OS) and Disease-Free Survival (DFS). Secondary outcomes of interest were the number of patients developing a second recurrence, the morbidity and major (Clavien Dindo ≥ III) morbidity rates and overall mortality. Data on patient demographics and baseline clinicopathologic characteristics of the involved hepatocellular carcinomas were also collected.

### 2.4. Risk of Bias Assessment

The risk of bias was independently analyzed by two authors (D.P, M.D.K) with a third author (D.I.T) acting as a referee for any disagreements. The risk of bias for non-randomized trials was evaluated using the ROBINS-I tool, which judges each study on the basis of seven criteria (bias due to confounding, bias due to selection of participants, bias in classification of interventions, bias due to deviations of intended interventions, bias due to missing data, bias in measurement of outcomes, bias in selection of the reported results). For each domain, the risk of bias can be low, moderate or serious. Concerning the Randomized Controlled Trials (RCTs), the revised Cochrane Collaboration RoB 2 tool was utilized, which incorporates five criteria instead of seven (bias arising from the randomization process, bias due to deviations from intended intervention, bias due to missing outcome data, bias in measurement of the outcome, bias in selection of reported result).

The Grading of Recommendations Assessment, Development, and Evaluation (GRADE) criteria was used by two reviewers (D.P and D.I.T.) to assess the overall quality of the evidence according to the involved risk of bias, inconsistency, indirectness and imprecision. The overall quality of evidence was categorized as very low, low, moderate and high.

### 2.5. Statistical Analysis

All statistical analyses in the present study were conducted using Stata v. 17 (StataCorp. 2021. Stata Statistical Software: Release 17. College Station, TX, USA: StataCorp LLC). Pooled Hazard Ratios (HRs) were calculated for the OS and DFS outcomes and Risk Ratios (RRs) for the second recurrence, morbidity, major morbidity and mortality outcomes using a predetermined Inverse Variance fixed effect model. Corresponding 95% Confidence Intervals (95% C.I.) were calculated and as per convention, were considered statistically significant if the data did not overlap with the value 1. Hazard Ratios were extracted directly from the study text, if available, or from the published

Kaplan-Meier survival curves using the “WebPlotDigitizer” software (https://automeris.io/WebPlotDigitizer, accessed on 20 August 2022) and the method described by Guyot et al. [21] Statistical heterogeneity was assessed with the Higgin’s I^2^ statistic; 0–30% values represented low heterogeneity, whereas values between 30–50% moderate heterogeneity, 50–75% substantial heterogeneity, and 75–100% considerable heterogeneity. For outcomes in which heterogeneity was measurable (I^2^ > 0%), a random effects (DerSimonian and Lair) was used, while in cases of non-existent heterogeneity (I^2^ = 0%) a fixed effect (inverse variance) model was selected instead.

Subgroup analysis was performed to assess whether adherence to the Milan criteria had any significant effects on the observed outcomes. Two subgroup analyses were performed; one regarding the reported patient selection criteria (i.e., “within the Milan criteria”, “outside the Milan criteria” and “patient selection criteria not reported”) and one regarding the type of study design with studies subdivided into prospective (i.e., randomized controlled and cohort studies), retrospective, or retrospective propensity score matched subgroups. Subgroup analysis was performed for outcomes that had at least two studies in each of the involved subgroups to allow for intergroup comparisons to be made. The presence of publication bias was explored visually by judging the symmetry of funnel plots as well as with Egger’s and Begg’s tests for every outcome incorporating at least ten studies. In all statistical analyses in the present study, a p-value below 0.05 was considered statistically significant.

## 3. Results

**Figure 1:**
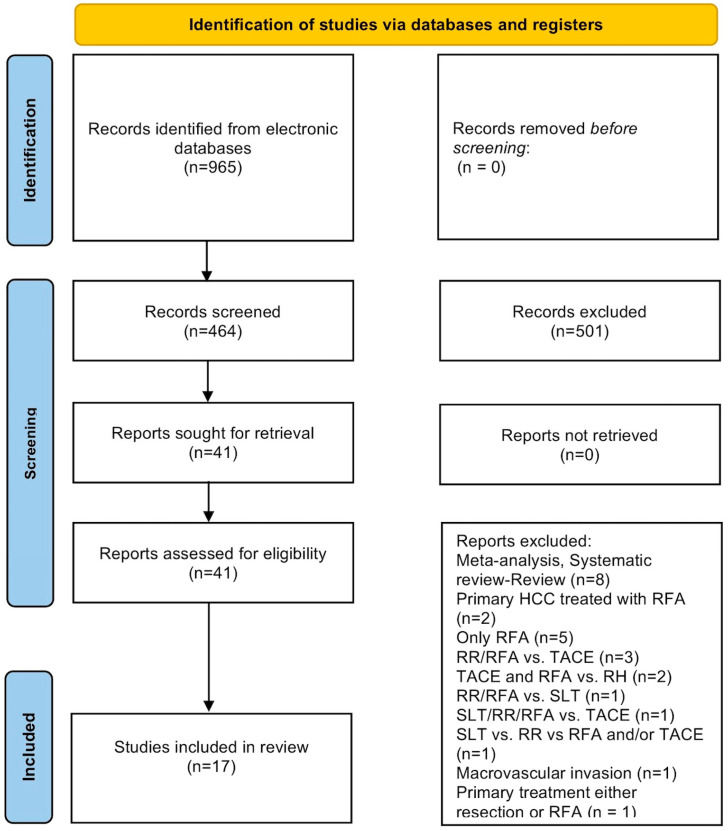
Search flow diagram.

Seventeen total studies, incorporating 2597 total patients (1203 in the RHR group versus 1394 in the RFA group), were deemed eligible for inclusion in the final data analysis (Figure 1) [22,23,24,25,26,27,28,29,30,31,32,33,34,35,36,37,38]. The majority of studies originated from East Asia (nine studies from China, three from Japan, one from Singapore, two from Taiwan and one from Korea), while one study was an international multicenter study. 

The included study dataset consisted of eight retrospective studies, six retrospective propensity score matched (PSM) studies, two prospective studies and one randomized controlled trial (Table 1). An overview of patient baseline characteristics is depicted in Table 2.

### 3.1. Critical Appraisal and Risk of Bias Assessment

The risk of bias assessment related to individual studies was summarized in the Supplemental Tables S1 and S2. For the non-randomized studies, the overall risk of bias was low in seven studies, moderate in four studies and serious in the remaining five studies. The risk of bias due to confounding was considered low in studies that used PSM, moderate in retrospective and prospective studies that reported on comparative baseline characteristics of the RHR and RFA cohorts, and serious in two studies that did not enclose any baseline patient characteristics. Serious bias due to patient selection was encountered in three studies with poor description of patient eligibility criteria. Moderate risk due to deviation of intended operations was encountered in two studies due to reported imbalances of the employed co-interventions along with RHR or RFA. Two studies excluded patients lost to follow-up from the analysis hence representing moderate risk for bias due to missing data, while one study reported more than 10% loss to follow-up and was considered to be at serious risk for missing data bias. Moderate risk for selective reporting bias was noted in a study that used a selective sub-cohort of HCC patients from a larger pool of potentially eligible patients. There were no issues in terms of intervention classification and outcome measurement biases due to the nature of involved interventions and outcomes. The single included randomized controlled trial was judged to be of low overall risk of bias. 

Regarding patient inclusion criteria, absence of extrahepatic spread, presence of intrahepatic HCC recurrence at a site distant from the original tumor and Child-Turcotte-Pugh (CTP) scores A or B were uniformly reported among the included studies. With respect to employed tumor number and size cut-offs, two studies [25,32] reported inclusion of patients with up to three tumors with a 3 cm size cut-off, while another two studies used 5 cm [38] and 6 cm cut-offs [36]. One study reported inclusion of BCLC stage 0 or A patients [31] and four studies adhered to the Milan criteria for size and number of tumors [23,24,30,33].

### 3.2. Primary Outcomes

Data on the Overall Survival were available in all seventeen included studies as shown in Table 3. 

**Figure 2:**
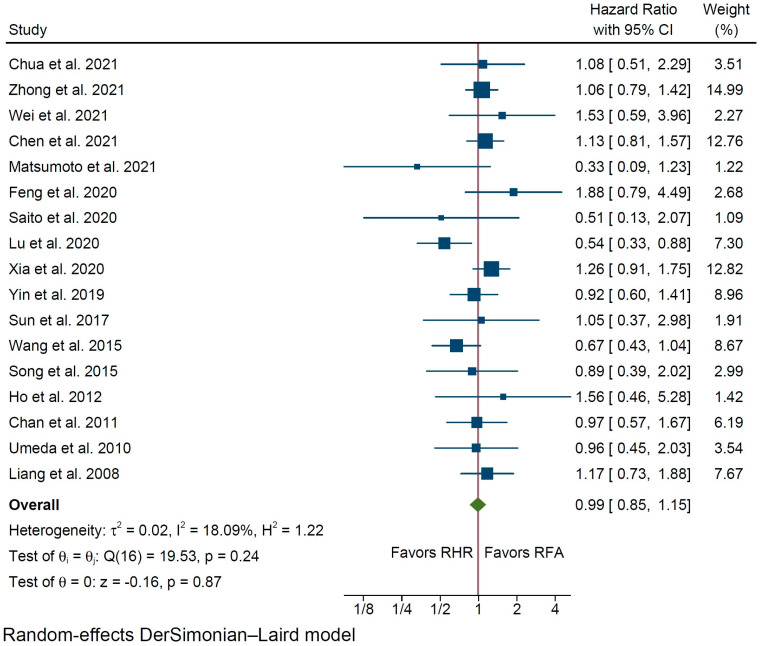
Forest plot for Overall Survival of patients undergoing repeat hepatic resection (RHR) versus radiofrequency ablation (RFA) of recurrent hepatocellular carcinoma [22,23,24,25,26,27,28,29,30,31,32,33,34,35,36,37,38].

**Figure 3:**
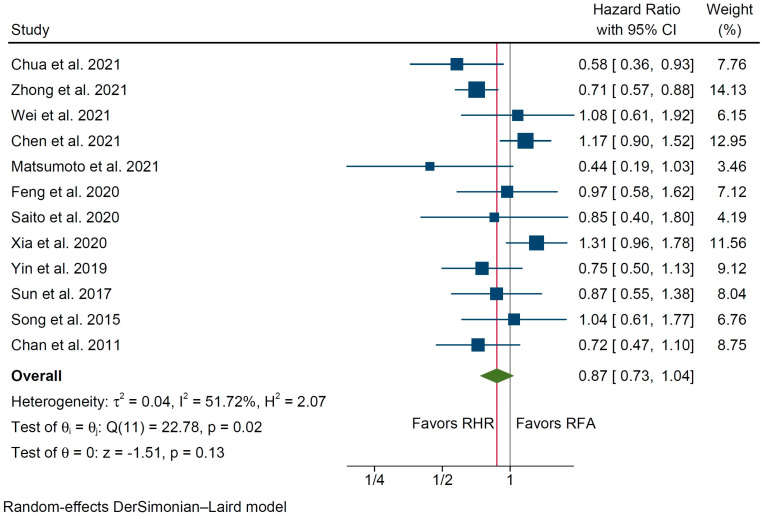
Forest plot for Disease-Free Survival of patients undergoing repeat hepatic resection (RHR) versus radiofrequency ablation (RFA) of recurrent hepatocellular carcinoma [22,23,24,25,26,27,28,30,31,32,34,36].

Pooled analysis did not reveal any statistically significant difference between the compared groups in terms of OS (HR 0.99% C.I. 0.85 to 1.15, *p* = 0.87, Figure 2), with low interstudy statistical heterogeneity (I^2^ = 18%). Disease-Free Survival was evaluated in 12 studies (Table 3), including 1746 total patients (851 in the RHR group vs. 895 in the RFA group), and was similar among the two groups (HR 0.87, 95% C.I. 0.73 to 1.04, *p* = 0.13, Figure 3) with substantial interstudy heterogeneity (I^2^ = 51.7%).

### 3.3. Secondary Outcomes

**Figure 4:**
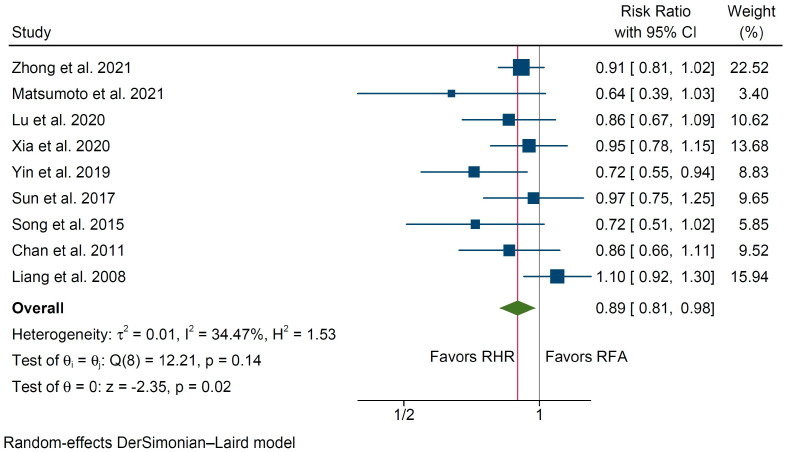
Forest plot of the odds for developing a second recurrence in patients undergoing repeat hepatic resection (RHR) versus radiofrequency ablation (RFA) of recurrent hepatocellular carcinoma [23,26,29,30,31,32,34,36,38].

The number of patients who developed subsequent HCC recurrence was evaluated in 9 studies totaling 1653 patients (723 in the RHR group vs. 930 in the RFA group, Table 3). Pooled analysis revealed decreased odds to develop a second HCC recurrence among patients undergoing RHR (RR 0.89, 95% C.I. 0.81 to 0.98, *p* = 0.02, Figure 4), with moderate interstudy heterogeneity (I^2^ = 34.5%).

**Figure 5:**
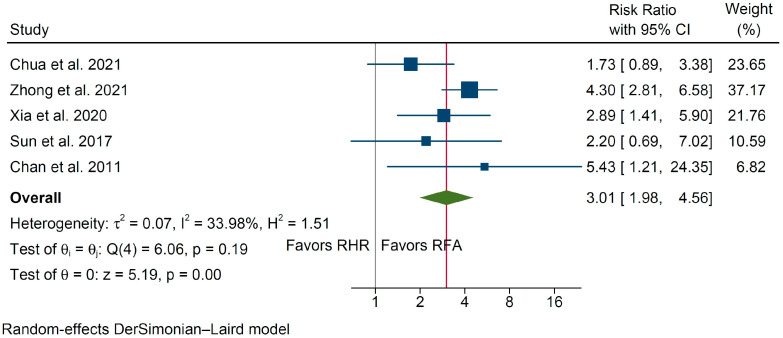
Forest plot of overall morbidity between the RHR and RFA groups [22,23,30,32,36].

Morbidity analysis on the basis of 5 studies reporting on 1363 patients (551 in the RHR group vs. 812 in the RFA group, Table 3) indicated that RHR was associated with increased morbidity (RR 3.01, 95% C.I. 1.98 to 4.56, *p* < 0.001, Figure 5) with moderate interstudy heterogeneity (I^2^ = 33.9%). 

**Figure 6:**
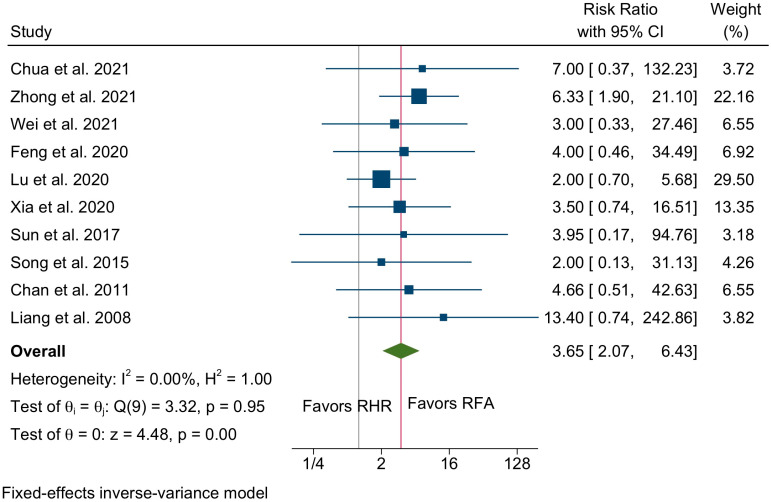
Forest plot of major morbidity between the RHR and RFA groups [22,23,24,27,29,30,32,34,36,38].

Major (Clavien Dindo ≥ III) morbidity was assessed in 10 studies incorporating 1850 patients (791 in the RHR group vs. 1059 in the RFA group). Pooled results indicate significantly increased major morbidity in patients undergoing RHR (RR 3.65, 95% C.I. 2.07 to 6.43, *p* < 0.001, Figure 6) with non-existent interstudy heterogeneity (I^2^ = 0%). 

**Figure 7:**
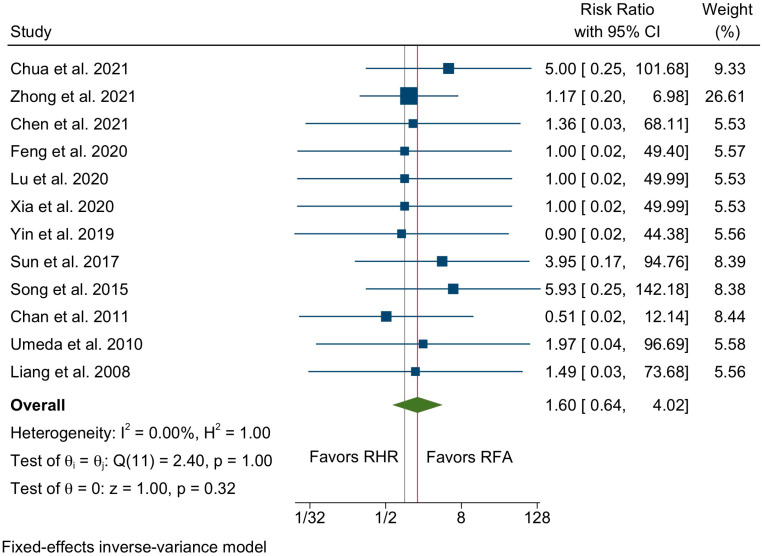
Forest plot of overall mortality between the RHR and RFA groups [22,23,25,27,29,30,31,32,34,36,37,38].

Data on post-procedural mortality were available in 12 studies with 2447 total patients (1024 in the RHR vs. 1423 in the RFA group, Table 3). Overall, six deaths were registered in the RHR group (0.5% mortality rate) and three in the RFA group (0.2% mortality rate), without any existing statistically significant difference between the two groups (RR 1.6, 95% C.I. 0.64 to 4.02, *p* = 0.32, Figure 7) and no interstudy heterogeneity (I^2^ = 0%). 

### 3.4. Subgroup Analysis

Subclassification of the included studies for the primary outcomes according to the adherence to the Milan criteria and study design type demonstrated that the observed HRs were comparable among the evaluated subgroups, with no statistically significant intergroup differences (supplementary Figures S1−S10). In general, studies not reporting any patient selection criteria reported more favorable OS, DFS and risk for second recurrence outcomes in the RHR group of patients versus studies with patients either within or outside the Milan criteria. None of the explored subgroups attained statistically significant results relative to OS and DFS, while only the subgroup not reporting any criteria exhibited statistical significance regarding the second recurrence outcome (supplementary Figures S1–S3).

Major morbidity and mortality were strongly associated with RHR across all subgroups, with the former being especially pronounced in the “within Milan criteria” subgroup (supplementary Figure S4) and the latter in the “no criteria” subgroup (supplementary Figure S5).

With respect to study design, retrospective and retrospective PSM studies generally exhibited equivalent pooled estimates, except for the major morbidity outcome in which retrospective studies exhibited a stronger association with major complications in RHR patients, albeit the subgroup was limited to two studies (supplementary Figure S9). Encountered statistical heterogeneity for the second recurrence outcome was exclusively attributable to retrospectively designed studies, while it did not exhibit any particular subgroup predilection in the remaining analyses.

### 3.5. Level of Evidence

The GRADE evaluation of the assessed outcomes resulted in moderate certainty for the Overall Survival outcome and low certainty for the remaining outcomes (Table 4). Risk of bias in the included studies was moderate based on the findings of the previously discussed risk of bias assessment. Inconsistency was moderate for the Disease-Free Survival and Morbidity outcomes due to the presence of moderate interstudy statistical heterogeneity (I^2^ = 47.4% and 33% respectively). Second recurrences, morbidity and mortality rates were secondary outcomes in the included studies and hence scored moderate for indirectness. Imprecision was considered serious in outcomes with patient sample sizes less than 400 and moderate in the remaining.

### 3.6. Publication Bias Assessment

Visual assessment of funnel plots did not reveal any substantial asymmetry for any of the analyzed outcomes (Supplementary Figures S11–S16). Evaluation for the presence of funnel plot asymmetry with Begg’s and Egger’s tests did not reveal any statistically significant findings relative to OS, DFS, mortality and major morbidity outcomes (Begg’s; *p* = 0.59, 1, 1, and 0.65, Egger’s; *p* = 0.48, 0.42, 0.76 and 0.95, respectively). The risk for publication bias was therefore low overall.

## 4. Discussion

The current analysis demonstrated that RFA is a safe and efficient alternative over RHR for selected patients with rHCC. In the absence of a structured algorithm for the management of patients with rHCC, repeat resection remains the treatment of choice, while RFA represents a feasible alternative with comparable short- and long-term outcomes. In fact, pooled analysis of the included studies did not reveal any statistically significant differences in terms of overall and disease-free survival between the two approaches. RFA was superior based on short-term safety outcome analysis. Specifically, RFA was associated lower overall, as well as major morbidity rates, however mortality was similar among patients who underwent RHR versus RFA. 

After primary margin free (R0) resection, 5-year recurrence still remains high with a reported incidence of approximately 60–70% [39]. Early recurrence occurs within 2 years and late recurrence after 2 years following primary treatment [40]. Recurrence of HCC occurs predominantly in the liver. While development of early intrahepatic recurrence has been associated with the performance of non-anatomical resections, resections with less than 1 cm free margin, unrecognized multifocal HCC, high serum AFP > 32 ng/mL and occult metastasis [40]. In contrast, late intrahepatic recurrence usually represents a de novo second primary tumor [41]. Other risk factors for intrahepatic recurrence include male sex, presence of underlying cirrhosis, multiple tumors, satellite nodules, maximum tumor size greater than 5 cm, microscopic and macroscopic vascular invasion [39]. Therefore, the presence of one or more risk factors highlights the need for close surveillance, for the early identification and treatment of these patients. According to the European Association for the Study of the Liver (EASL) guidelines, several tests can be implemented for surveillance among patients with high risk for recurrence [14]. However, only ultrasound demonstrates acceptable specificity (>90%), sensitivity (ranging from 58–89%) and cost effectiveness compared with other surveillance tests when performed in a six-month interval. Serum AFP, though widely used as a biomarker for the diagnosis of HCC, lacks specificity for patient surveillance particularly in cirrhotic patients with viral infection or underlying liver disease [14,42]. 

The main advantages of RFA relate to its lower complication rates. As a less invasive technique it minimizes the perioperative stress, which can even be diminished if performed percutaneously for easily accessible hepatic lesions. Moreover, it causes minor damage to the surrounding healthy liver parenchyma, thus preserving the maximum liver remnant [19], in the setting of a small or cirrhotic liver. It is also possible to perform in tumors located deep inside the liver parenchyma without resulting in a disproportionate transection plane to approach those tumors. These advantages provide the rationale for RFA for recurrent HCC. The technical limitations of RFA include the challenge to provide a 3-dimensional ablation margin, as well as the relative limitations related to tumor size [43]. Furthermore, RFA may be associated with potential risk of tumor seeding along the electrode’s track and potentially dangerous thermal injury when performed near a large vessel or liver capsule [44]. As demonstrated in the current analysis, length of hospital stay and estimated blood loss were lower among patients undergoing RFA versus RHR, perhaps as expected in patients given the challenges of repeat resection in the setting of multiple adhesions. 

While RFA was associated with better short-term outcomes, RHR was associated with lower re-recurrence versus RFA. Resection of an adequate margin length can protect against a possible recurrence, as the resected segment may contain potential microscopic metastases or microvascular invasion sites not pre- or intraoperatively recognized. Nonetheless, performance of RHR for rHCC, similar to resection for primary HCC, remains highly dependent on tumor size and location, patient overall fitness and even more importantly liver function [8]. Specifically for cirrhotic patients, although a minor or major resection may be technically feasible, it may not be well tolerated by the patient due to inadequate future liver volume or function thus making RFA an attractive alternative treatment option. As with primary HCC and even more importantly in the absence of structured guidelines, multidisciplinary team decision-making is fundamental to treating patients with recurrent HCC. For patients with good performance status, adequate hepatic reserve and a lesion that is technically resectable, RHR may be a good option. However, for patients who do not meet these criteria, thermal ablation may be a strategy that has reasonable results, as demonstrated by the data in the current meta-analysis.

Several previously published meta-analyses, with a more limited number of included studies, reported similar outcomes to the current study [45,46]. Notably, these analyses were somewhat flawed by the fact that previous authors included studies in which patients had their primary HCC managed with resection or ablation [47]. In contrast, we only included only studies with patients who had the primary HCC treated solely with resection, thus justifying the term RHR. There were, however, several limitations inherent to the current study. We included studies published only in English language thus a number of non-English studies relevant may have been missed. The large number of included non-randomized retrospective studies pose an inevitable risk of selection bias. Additionally, the majority of the included studies derived from Asian countries possibly limiting the generalizability of outcomes to other patient populations. Information about the status of the primary HCC relative to the subsequent recurrence including surgical margin status, type of resection (anatomical or non-anatomical), tumor grade or microvascular invasion most probably were highly heterogeneous among the included studies. RFA has several technical limitations, rendering its use in difficult areas of the liver (near large vessels, or near capsule). Poor liver function (previous chemotherapy, cirrhosis), or poor patient status, may also have led toward a more conservative therapeutic approach in this subset of patients. Moreover, recurrence might have been influenced by the initial surgical approach and a variety of risk factors including molecular profiling, quality of liver parenchyma, thus altering second recurrence rates and long-term outcomes. Finally, while many centers now use microwave ablation rather than RFA, data from the current study are likely applicable to other thermal ablative approaches.

## 5. Conclusions

In conclusion, RFA is a safe and efficient alternative to RHR for selected patients with recurrent HCC, especially patients who are not candidates for RHR. RFA was associated with low peri-procedural complications and reasonable long-term outcomes. RHR resection remains, however, the preferred treatment option for patients with good performance status, adequate future liver remnant and function, whenever feasible, as RHR was associated with better long-term local disease control. Emerging biomarkers may have a role in stratification patients relative to genetic profiles, which in turn may help identify patient populations that may benefit more from ablation versus resection.

## Figures and Tables

**Table 1:** Characteristics of the included studies (RHR vs. RFA).

Author; Year	Country	Type of Study	n Patients	Age	Sex (F) (%)
Chua; 2021 [22]	Singapore	Retrospective, PSM	52 vs. 52	63.5 (56.5–71.0) * vs. 62.5 (56.5–66) *	45 (92.6) vs. 46 (88.5)
Zhong; 2021 [23]	China	Retrospective, PSM	227 vs. 227	N/a	33 (14.6) vs. 36 (15.9)
Wei; 2021 [24]	China	Retrospective, PSM	35 vs. 35	N/a	4 (11.4) vs. 4 (11.4)
Chen; 2021 [25]	China	Retrospective	138 vs. 138	50.7 ± 10.5 vs. 49.2 ± 10.9	13 (9.4) vs. 16 (8.5)
Matsumoto; 2021 [26]	Japan	Retrospective	23 vs. 11	66 (55–84) * vs. 67 (42–79)	3 (13.1) vs. 0 (0)
Feng; 2020 [27]	Multicenter	Retrospective, PSM	48 vs. 48	56.6 ± 9.2 vs. 58.2 ± 7.5	7 (4.6) vs. 6 (2.5)
Saito; 2020 [28]	Japan	Retrospective	17 vs. 26	N/a	N/a
Lu; 2020 [29]	China	Retrospective, PSM	120 vs. 120	50.9 ± 11.6 vs. 50.3 ± 10.3	16 (3.4) vs. 12 (10)
Xia; 2020 [30]	China	RCT	120 vs. 120	50 (24–58) * vs. 52 (25–59) *	13 (10.9) vs. 11 (9.2)
Yin; 2019 [31]	China	Retrospective	57 vs. 51	57 ± 12 vs. 60.26 ± 9.5	16 (28.1) vs. 20 (39.2)
Sun; 2017 [32]	Taiwan	Retrospective	43 vs. 57	60 (35–76) * vs. 63 (27–81) *	9 (21) vs. 19 (33.4)
Wang; 2015 [33]	China	Prospective	128 vs. 162	50.2 ± 10.1 vs. 51 ± 10.1	15 (11.8) vs. 14 (8.7)
Song; 2015 [34]	Korea	Retrospective, PSM	39 vs. 78	52.5 ± 9.8 vs. 53.6 ± 10.9	8 (20.5) vs. 20 (25.7)
Ho; 2012 [35]	Taiwan	Retrospective	54 vs. 50	56.3 ± 12.3 vs. 61 ± 11.1	14 (25.9) vs. 11 (22)
Chan; 2011 [36]	China	Prospective	29 vs. 45	52 (38–79) * vs. 59 (36–80) *	N/a
Umeda; 2010 [37]	Japan	Retrospective	29 vs. 58	64.8 ± 0.79	N/a
Liang; 2008 [38]	China	Retrospective	44 vs. 66	48.8 ± 12.0 vs. 54.6 ± 10.8	5 (11.4) vs. 12 (18.2)

RHR; repeat liver resection, RFA; radiofrequency ablation, PSM; propensity score matching, RCT; randomized controlled trial, N/A not available, * Results presented as median (range).

**Table 2:** Baseline patient characteristics in the included studies.

Author; Year	CTP A/B	Single Nodule	Tumor Size (cm)	AFP (ng/mL)	HBV Infection	Vascular Involvement
*RHR* vs. *RFA, n (%)*						
Chua; 2021 [22]	45 (90)/7(10) vs. 49 (96)/2 (4)	38 (74.5) vs. 43 (82.7)	3.0 (2.0–4.5) vs. 2.9 (2.0–4.0) *	12 (5–42) vs. 14(4–75) *	31 (63.3%) vs. 41 (80.4%)	5 (9.6%) vs. 9 (18%)
Zhong; 2021 [23]	222 (97.8)/5 (2.2) vs. 224 (98.7)/3 (1.3)	171 (75.3) vs. 172 (75.7)	≥3 cm; 99 (43.6) vs. 92(40.5) <3 cm; 128 (56.4) vs. 135 (59.5)	≥200; 45 (19.8) vs. 46 (20.2) <200; 182 (80.2) vs. 181 (79.7)	193 (85) vs. 192 (84.5)	N/a
Wei; 2021 [24]	35 (100)/0 vs. 35 (100)/0	24(68.6%) vs. 30(85.7%)	≥3 cm; 3 (8.6) vs. 2 (5.7) <3 cm; 32 (91.4) vs. 3 (94.3)	≥200; 12 (34.3) vs. 7 (2) <200; 23 (65.7) vs. 28 (80)	N/a	N/a
Chen; 2021 [25]	N/a	119 (86.2) vs. 148 (78.7)	2.4 ± 0.5 vs. 2.2 ± 0.4	>20; 91 (65.9) vs. 127 (67.5) ≤20; 47 (34.1) vs. 61(32.5)	117 (84.7) vs. 145 (77.1)	32 (23.2) vs. 59 (31.4)
Matsumoto; 2021 [26]	22 (95.6)/1 (4.4) vs. 9 (81.8)/2 (18.2)	19 vs. 8	3.2 (0.9–10.5) vs. 2 (1.5–9.6)	N/a	7 (30.4) vs. 2 (18.1)	N/a
Feng; 2020 [27]	45 (93.8)/3 (6.2) vs. 41 (85.4)/7 (4.6)	37 (77) vs. 34 (70.8)	2.5 (2–3) vs. 2.5 (2–3.3) *	13.1 (2.8–133.1) vs. 6.1 (2.4–182.1) *	48 (100) vs. 48 (100)	N/a
Saito; 2020 [28]	N/a	N/a	N/a	N/a	N/a	N/a
Lu; 2020 [29]	120 (100)/0 vs. 120 (100)/0	106 (88.3) vs. 106 (88.3)	2.2 ± 1 vs. 2.4 ± 1.1	>20; 50 (41.6) vs. 45 (37.5) ≤20; 70 (58.4) vs. 75 (62.5)	108 (90) vs. 112 (93.3)	N/a
Xia; 2020 [30]	120 (100)/0 vs. 120 (100)/0	99 (82.5) vs. 93 (77.5)	4 (1–5) vs. 4 (1.1–11.2) *	>200; 70 (58.3) vs. 73 (60.8) ≤200; 50 (41.7) vs. 47 (39.2)	98 (81.6) vs. 91 (75.8)	38 (31.6) vs. 35 (29.1)
Yin; 2019 [31]	55 (96.5)/2 (3.5) vs. 46 (90.1)/5 (9.9)	52 (91.2) vs. 48 (94.1)	3.2 ± 2.5 vs. 2.6 ± 0.9	167.9 ± 357.2 vs. 266.3 ± 420.2	53 (92.9) vs. 48 (94.1)	N/a
Sun; 2017 [32]	35 (97.2)/1 (2.8) vs. 50 (100)/0	N/a	3.9 (1.0–16.0) vs. 3.9 (1.3–15.0) *	602 (1–11681) vs. 1090 (3–29141)	21 (48.8) vs. 32 (56.1)	8 (18.6) vs. 7 (12.3)
Wang; 2015 [33]	N/a	89 (69.5) vs. 107 (66)	2.4 ± 0.9 vs. 2.3 ± 0.7	>20; 56 (43.7) vs. 77 (47.5) ≤20; 72 (56.3) vs. 85 (52.5)	119 (92.9) vs. 142 (87.6)	23 (17.9) vs. 0
Song; 2015 [34]	39 (100)/0 vs. 78 (100)/0	32 (82) vs. 65 (83.3)	>2 cm; 17 (43.6) vs. 31 (39.7) ≤2 cm; 22 (56.4) vs. 57 (60.3)	>200; 6 (15.4) vs. 9 (11.5) ≤200; 33 (84.6) vs. 69 (88.5)	36 (92.3) vs. 70 (89.7)	15 (38.5) vs. 27 (34.6)
Ho; 2012 [35]	51 (94.4)/2 (3.7) vs. 50 (100)/0	N/a	2.9 ± 1.8 vs. 2.3 ± 1.9	>400; 10 (18.5) vs. 7 (14)	39 (72.2) vs. 27 (54)	4 (7) vs. 0
Chan; 2011 [36]	29 (100)/0 vs. 40 (88.8)/5 (11.2)	N/a	3.5 (1.0–14.5) vs. 5.5 (1.5–22.0) *	64 (2–167,138) vs. 90 (1–197,122) *	26 (89.6) vs. 40 (88.8)	N/a
Umeda; 2010 [37]	28 (96.5)/1 (3.5) vs. 54 (93.1)/4 (6.9)	18 (62) vs. 34 (58.6)	4.3 ± 0.55 vs. 3.2 ± 0.39	≥100; 7 (34.2) vs. 9 (15.5) <100; 22 (75.8) vs. 49 (84.5)	8 (27.5) vs. 11 (18.9)	9 (31) vs. 18 (31)
Liang; 2008 [38]	44 (100)/0 vs. 64 (96.9)/2 (3.1)	34 (77.2) vs. 48 (72.7)	≤3 cm; 26 vs. 44 >3 cm; 18 vs. 22	≤400; 30 (59) vs. 52 (78.8) >400; 14 (41) vs. 14 (21.2)	41 (93.2) vs. 60 (90.9)	N/a

CTP; Child Turcotte Pugh score, AFP; alpha fetoprotein, HBV, Hepatitis B virus, RHR; repeat liver resection, RFA; radiofrequency ablation, N/a; not available * Results presented as median (range or IQR).

**Table 3:** Summary of individual study findings.

Author; Year	3-Year OS	5-Year OS	3-Year DFS	5-Year DFS	Second Recurrence	Morbidity	CD ≥ III Morbidity	Mortality
*RHR* vs. *RFA*, *n* (%)								
Chua; 2021 [22]	72.5% vs. 62.6%	71.3% vs. 65.7%	N/a	63.2% vs. 78.9%	N/a	18 (34.6) vs. 10 (20)	3 (6) vs. 0	2 (3.8) vs. 0
Zhong; 2021 [23]	67.4% vs. 71.3%	56.4% vs. 53.1%	37.5% vs. 28.1%	25.5% vs. 16%	155 (68.2) vs. 170 (74.8)	66 (21.5) vs. 27 (5)	19 (6.2) vs. 3 (0.5)	2 (0.6) vs. 3 (0.5)
Wei; 2021 [24]	59% vs. 71.4%	N/a	32.3% vs. 34%	N/a	N/a	N/a	3 (8.6) vs. 1 (2.9)	N/a
Chen; 2021 [25]	N/a	N/a	N/a	N/a	N/a	N/a	N/a	None
Matsumoto; 2021 [26]	89.% vs. 74%	84.9% vs. 74%	43.4% vs. 15.4%	43.4% vs. 0	12 (52.2) vs. 9 (81.8)	N/a	N/a	N/a
Feng; 2020 [27]	70.3% vs. 67%	38.7% vs. 60.3%	25.9% vs. 32.8%	21.6% vs. 9.8%	N/a	N/a	4 (8.3) vs. 1 (2)	None
Lu; 2020 [29]	81.5% vs. 61%	71.8% vs. 41.7%	N/a	N/a	59 (49.1) vs. 69 (57.5)	N/a	10 (8.3) vs. 5 (4.1)	None
Xia; 2020 [30]	65.8% vs. 52.5%	43.6% vs. 38.5%	52.4% vs. 41.7%	36.2% vs. 30.2%	73 (60.8) vs. 77 (64.2)	26 (21.6) vs. 9 (7.5)	7 (5.8) vs. 2 (1.6)	None
Yin; 2019 [31]	50.5% vs. 50.9%	29.7% vs. 26%	39.4% vs. 32.8%	26.6% vs. 20.4%	32 (56.1) vs. 40 (78.4)	N/a	N/a	None
Sun; 2017 [32]	82.7% vs. 77.2%	56.4% vs. 52.6%	32.1% vs. 26.6%	28.6% vs. 16.6%	30 (69.7) vs. 41 (71.9)	7 (16.3) vs. 4 (7)	1 (2.3) vs. 0	1 (2.3) vs. 0
Wang; 2015 [33]	84.1% vs. 73.4%	64.5% vs. 37%	N/a	N/a	N/a	N/a	N/a	N/a
Song; 2015 [34]	88.8% vs. 85.7%	83.9% vs. 72.1%	48.5% vs. 45.1%	43.1% vs. 39.4%	18 (47.3) vs. 117 (65.7)	N/a	1 (2.5) vs. 1 (1.2)	1 (2.5) vs. 0
Ho; 2012 [35]	N/a	72% vs. 83%	N/a	N/a	N/a	N/a	N/a	N/a
Chan; 2011 [36]	56.5% vs. 68.2	35.2% vs. 44.5%	24.2% vs. 12.4%	24.2% vs. 9.3%	21 (72.4) vs. 38 (84.4)	7 (24.1) vs. 2 (4.4)	3 (10.3) vs. 1 (2.2)	0 vs. 1 (2.2)
Umeda; 2010 [37]	66.8% vs. 75.1%	56.1% vs. 48.3%	N/a	N/a	N/a	N/a	N/a	None
Liang; 2008 [38]	44.5% vs. 48.6%	27.6% vs. 39.9%	N/a	N/a	38 (86.3) vs. 52 (78.7)	N/a	N/a	None

RHR; repeat liver resection, RFA; radiofrequency ablation, N/a; not available.

**Table 4:** Results of Quality Assessment of Studies Included in Meta-Analyses (The Grading of Recommendations Assessment, Development and Evaluation–GRADE).

Outcome	n Studies	Study Design	Risk of Bias	Inconsistency	Indirectness	Imprecision	Others	Certainty
Overall Survival	17	Observational studies, n = 16 RCT, n = 1	Moderate	Low	Low	Moderate	None	⊕⊕⊕☐ Moderate
Disease-Free Survival	12	Observational studies, n = 11 RCT, n = 1	Moderate	Moderate	Low	Moderate	None	⊕⊕☐☐ Low
Second Recurrence	9	Observational studies, n = 9 RCT, n = 1	Moderate	Moderate	Moderate	Moderate	None	⊕⊕☐☐ Low
Morbidity	5	Observational studies, n = 4 RCT, n = 1	Moderate	Moderate	Moderate	Serious	None	⊕☐☐☐ Very Low
Morbidity, CD≥III	10	Observational studies, n = 9 RCT, n = 1	Moderate	Low	Moderate	Moderate	None	⊕⊕☐☐ Low
Mortality	12	Observational studies, n = 11 RCT, n = 1	Moderate	Low	Moderate	Moderate	None	⊕⊕☐☐ Low

RCT; randomized controlled trial, CD; Clavien-Dindo classification; ⊕: Achieving one level of quality of evidence; ☐: Decline in one level of quality of evidence.

## Supplementary Materials

The following supporting information can be downloaded at: https://www.mdpi.com/article/10.3390/cancers14215398/s1, Figure S1: Subgroup analysis forest plot for overall survival, Figure S2: Subgroup analysis forest plot for disease-free survival, Figure S3: Subgroup analysis forest plot for second recurrence, Figure S4: Subgroup analysis forest plot for ≥CDIII morbidity, Figure S5: Subgroup analysis forest plot for mortality, Figure S6: Subgroup analysis by study design, forest plot for OS, Figure S7: Subgroup analysis by study design, forest plot for DFS, Figure S8: Subgroup analysis by study design, forest plot for second recurrence, Figure S9: Subgroup analysis by study design, forest plot for ≥CDIII morbidity, Figure S10: Subgroup analysis by study design, forest plot for mortality, Figure S11: Funnel plot of studies included in the overall survival analysis, Figure S12: Funnel plot of studies included in the disease-free survival analysis, Figure S13: Funnel plot of studies included in the second recurrence analysis, Figure S14: Funnel plot of studies included in the morbidity analysis, Figure S15: Funnel plot of studies included in the ≥CDIII morbidity analysis, Figure S16: Funnel plot of studies included in the mortality analysis, Table S1: Risk of bias summary for non-randomized studies using the ROBINS-I tool, Table S2. Risk of bias summary for randomized controlled trials using the RoB 2 tool.
