# Supplementary material for: Meta-Analysis of Repeat Hepatectomy versus Radiofrequency Ablation for Recurrence of Hepatocellular Carcinoma
Source: Cancers (Basel). 2022 Nov 2;14(21):5398. doi: 10.3390/cancers14215398 (PMC9655365; doi:10.3390/cancers14215398)
Supplement: Supplementary file 1 [file cancers-14-05398-s001.zip › cancers-1962701-supplementary.pdf]

Supplementary Figures

Supplementary Figure S1. Subgroup analysis forest plot for overall survival

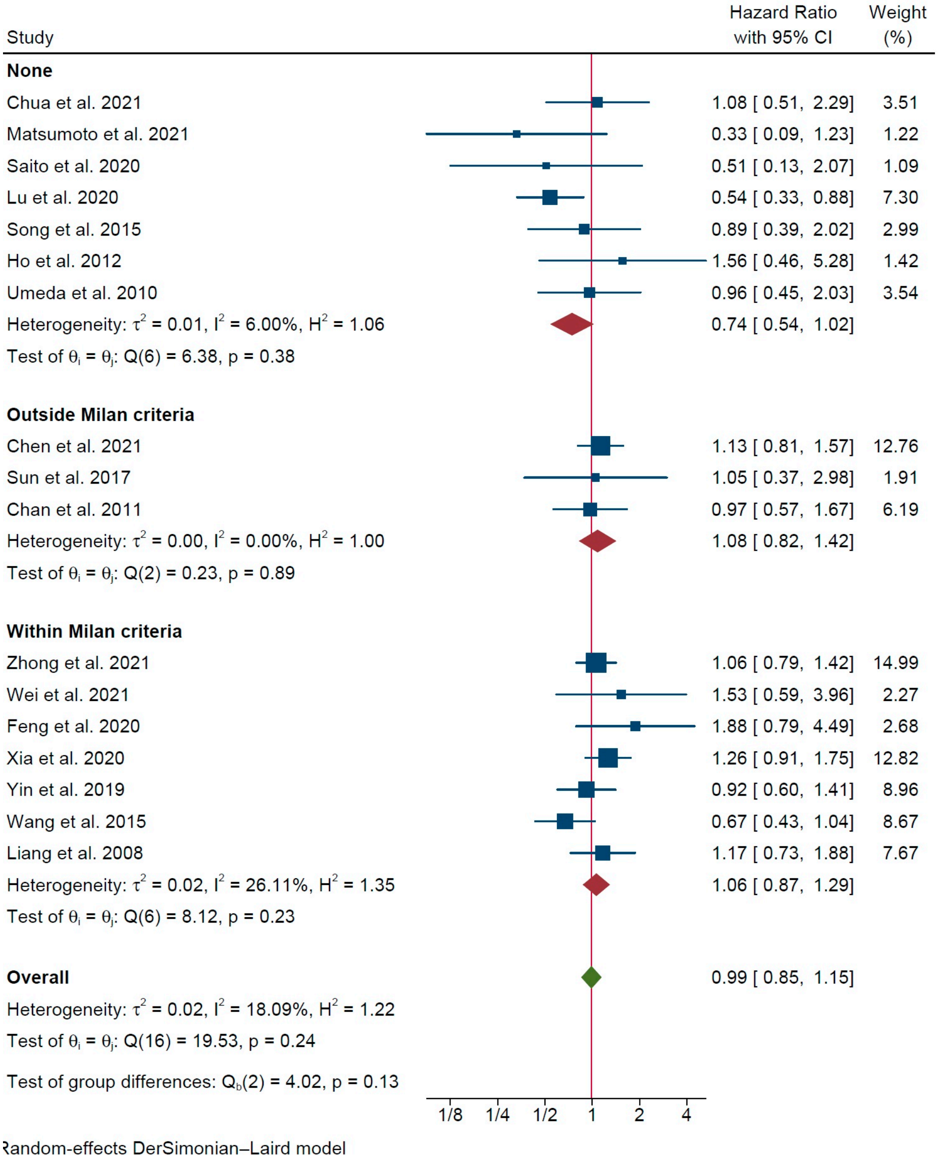

Supplementary Figure S2. Subgroup analysis forest plot for disease-free survival.

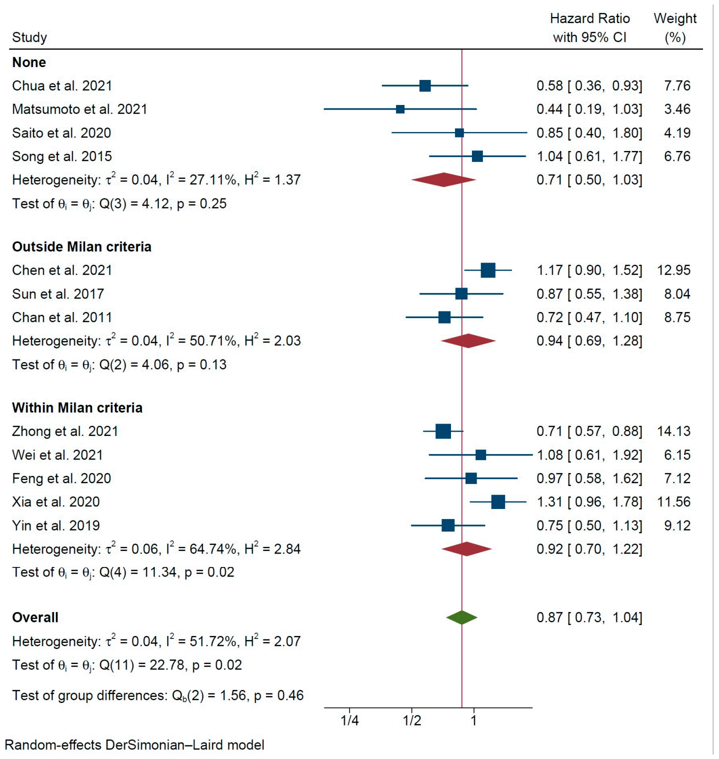

Supplementary Figure S3 Subgroup analysis forest plot for second recurrence.

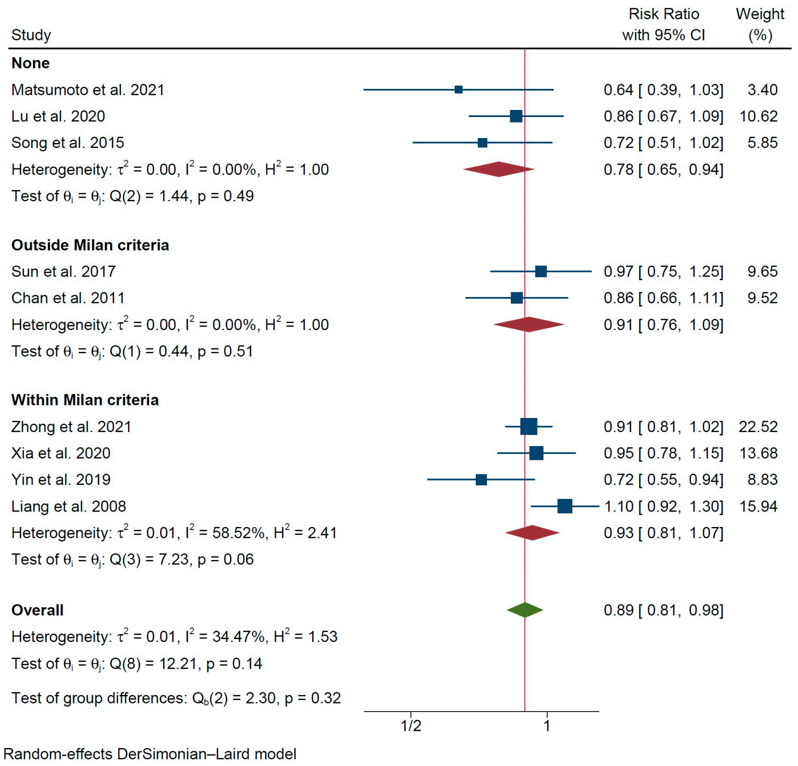

Supplementary Figure S4. Subgroup analysis forest plot for ≥CDIII morbidity.

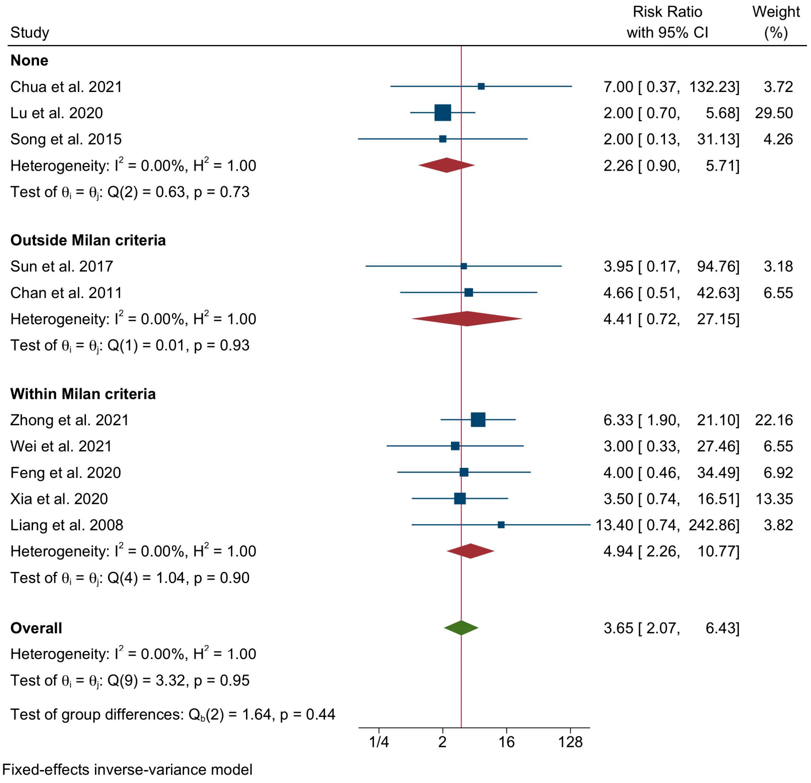

Supplementary Figure S5. Subgroup analysis forest plot for mortality.

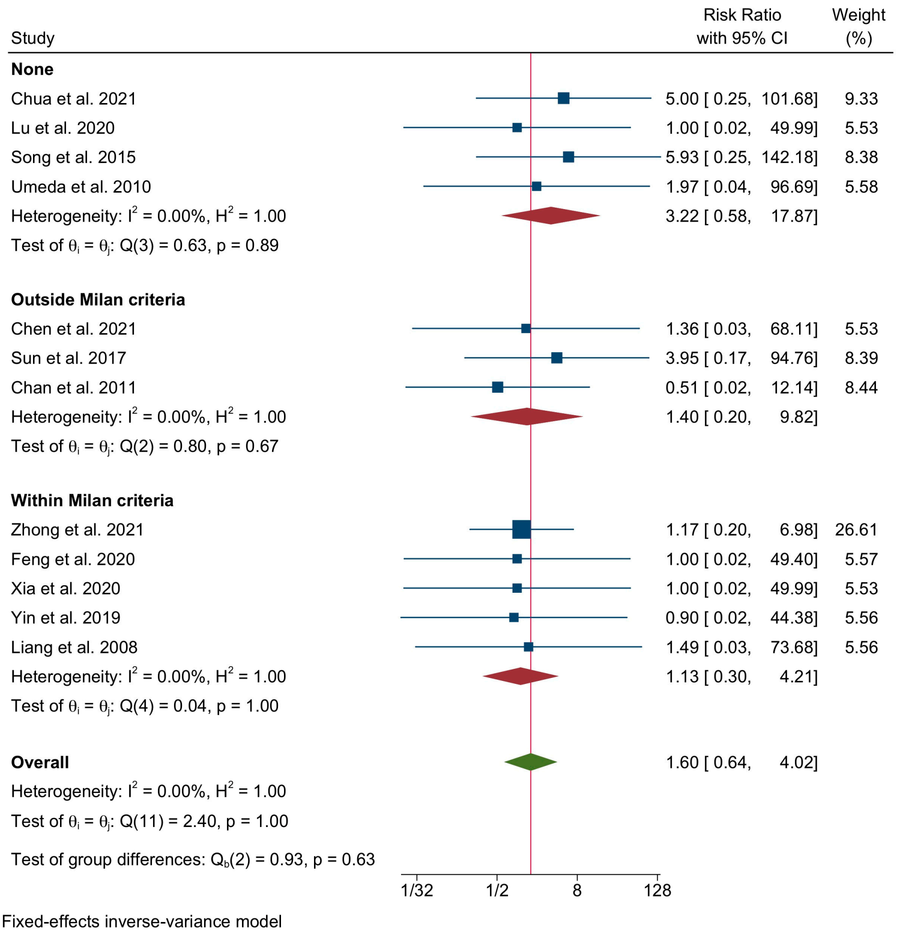

Supplementary Figure S6. Subgroup analysis by study design, forest plot for OS.

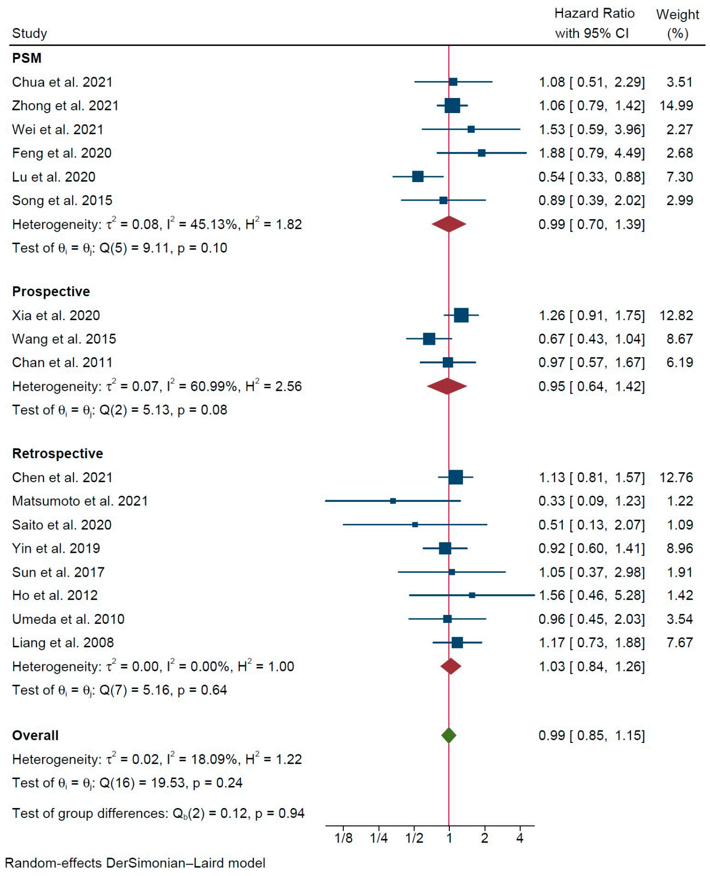

Supplementary Figure S7. Subgroup analysis by study design, forest plot for DFS.

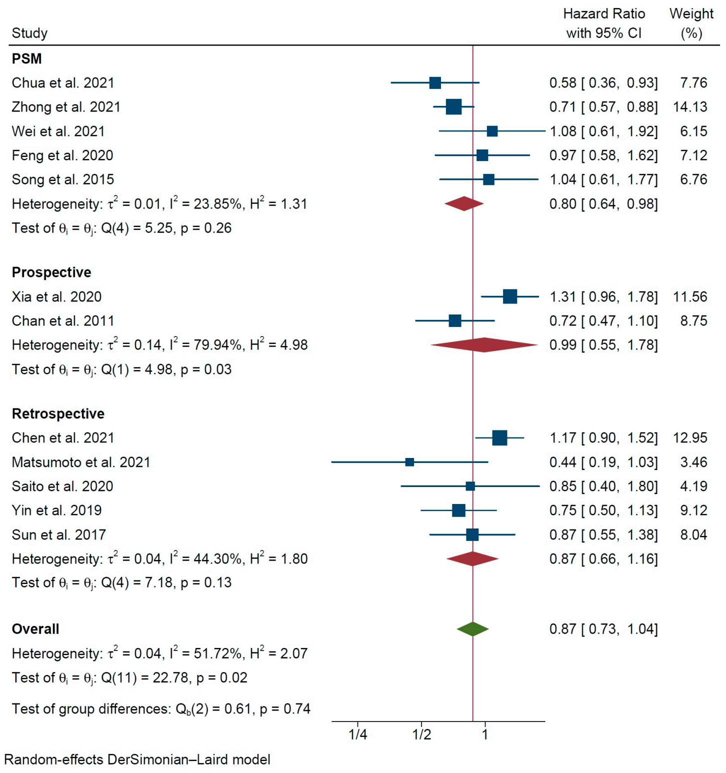

Supplementary Figure S8. Subgroup analysis by study design, forest plot for second recurrence.

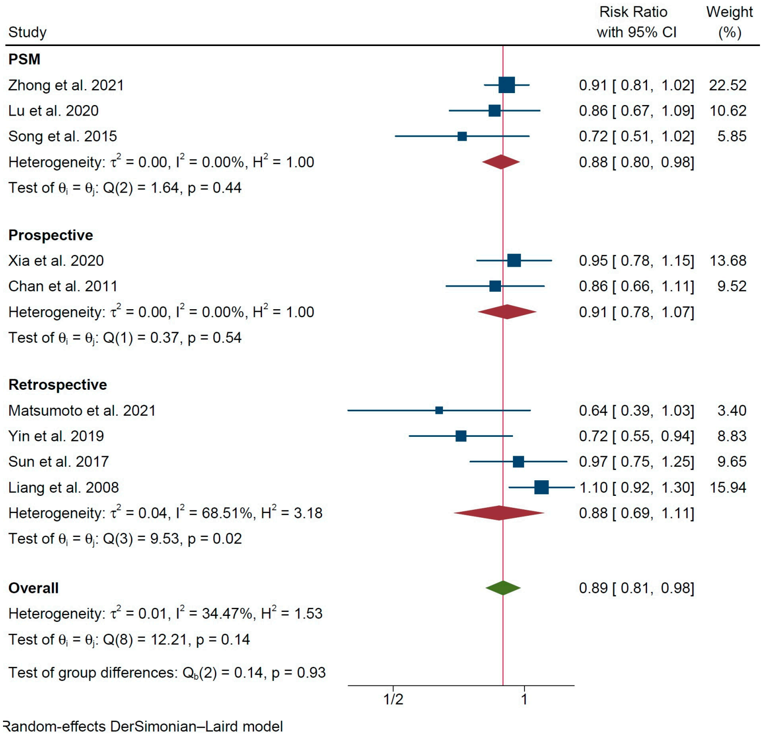

Supplementary Figure S9. Subgroup analysis by study design, forest plot for ≥CDIII morbidity.

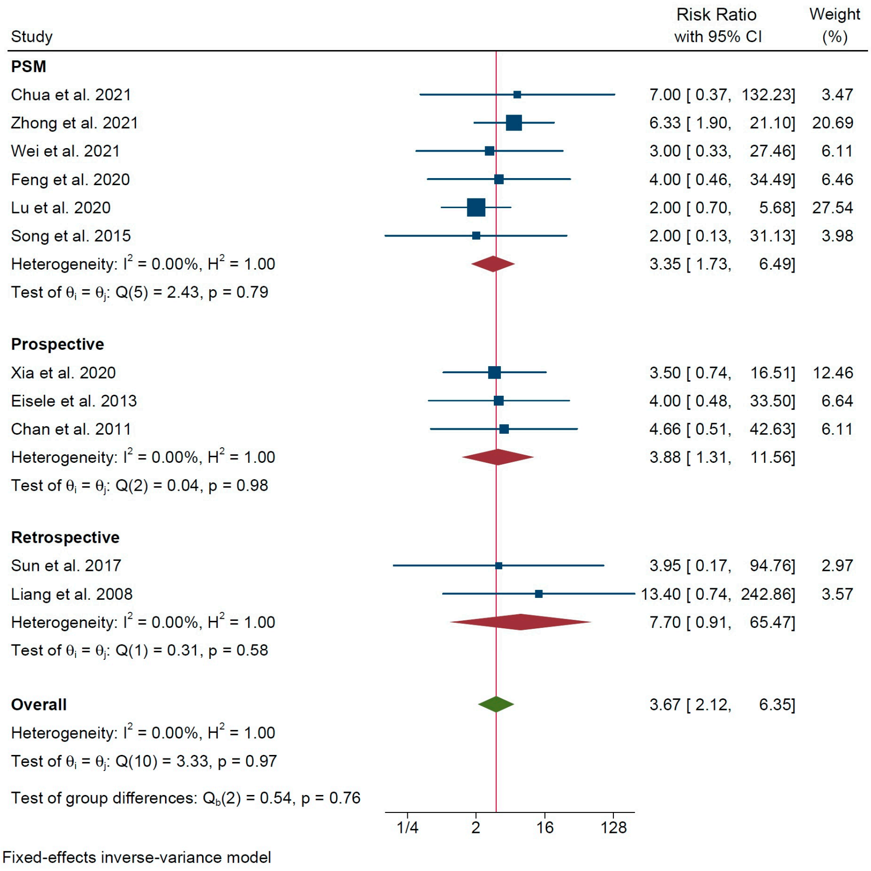

Supplementary Figure S10. Subgroup analysis by study design, forest plot for mortality.

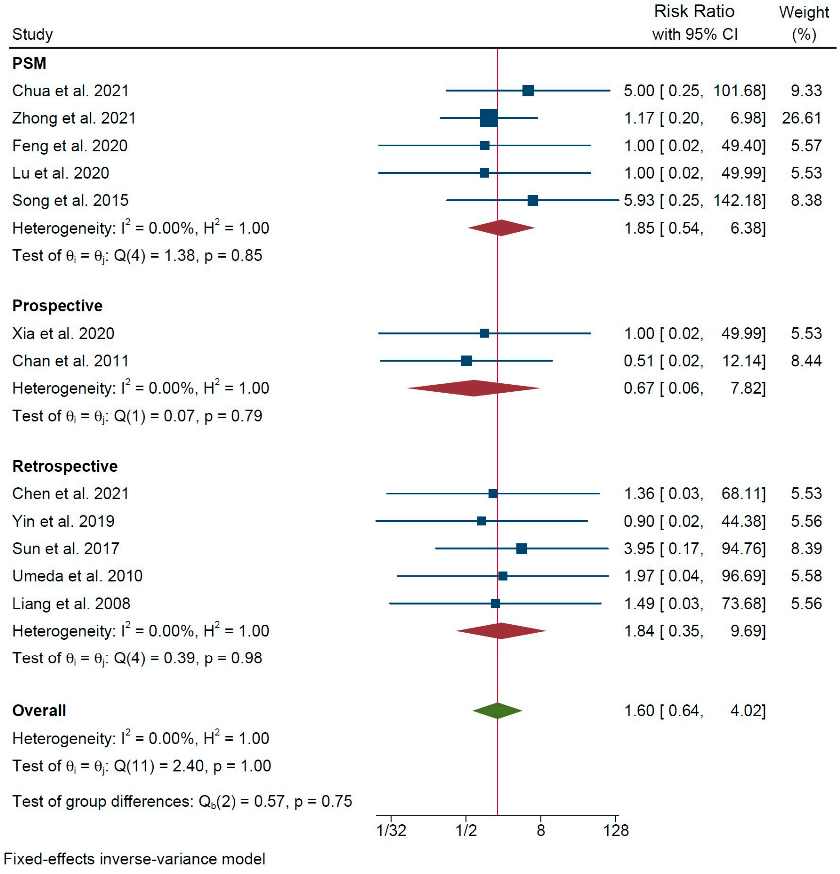

Supplementary Figure S11. Funnel plot of studies included in the overall survival analysis.

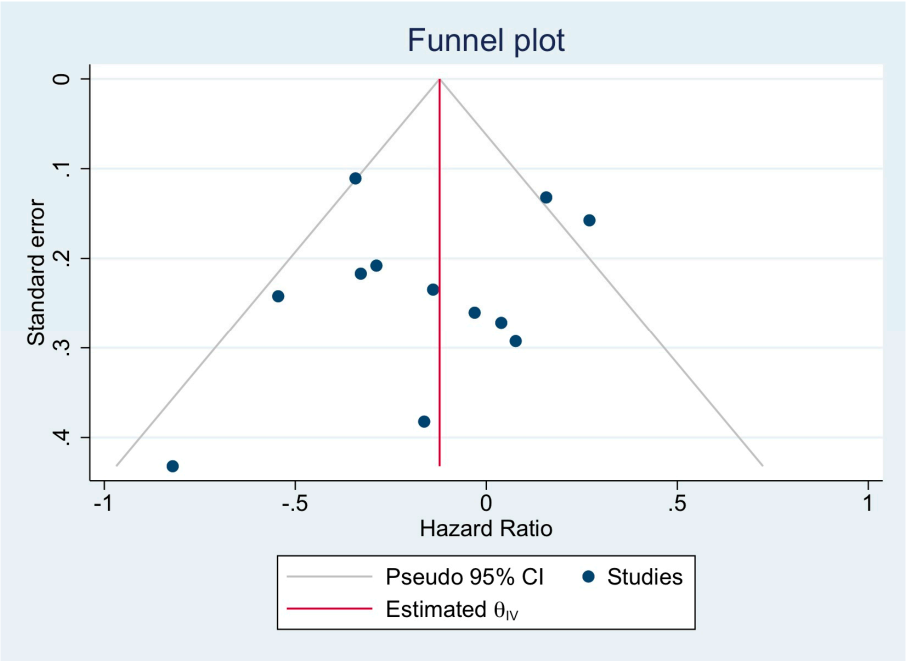

Supplementary Figure S12. Funnel plot of studies included in the disease-free survival analysis.

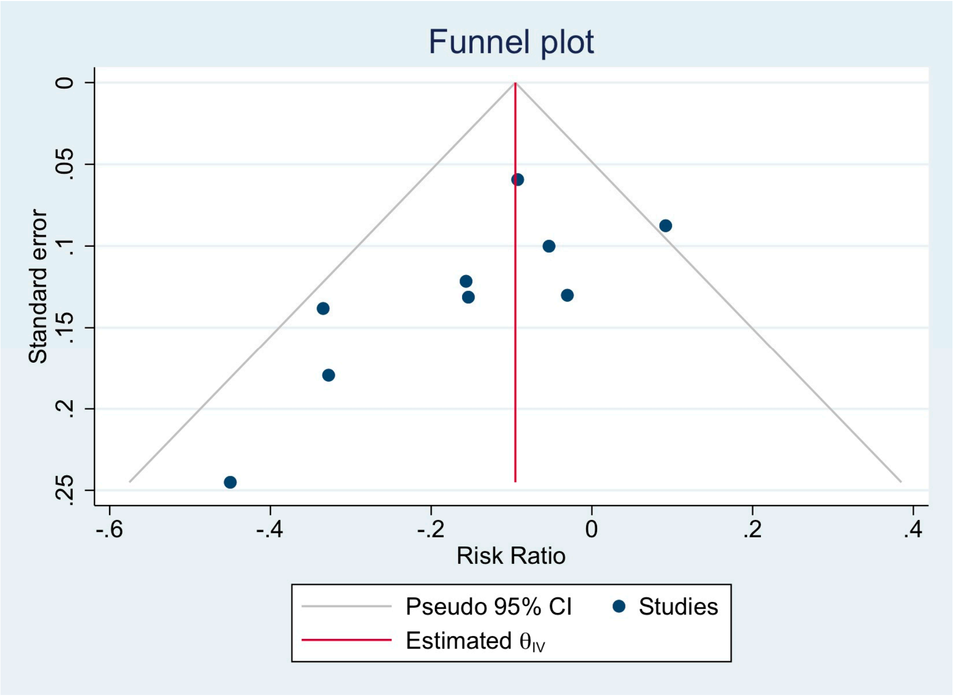

Supplementary Figure S13. Funnel plot of studies included in the second recurrence analysis.

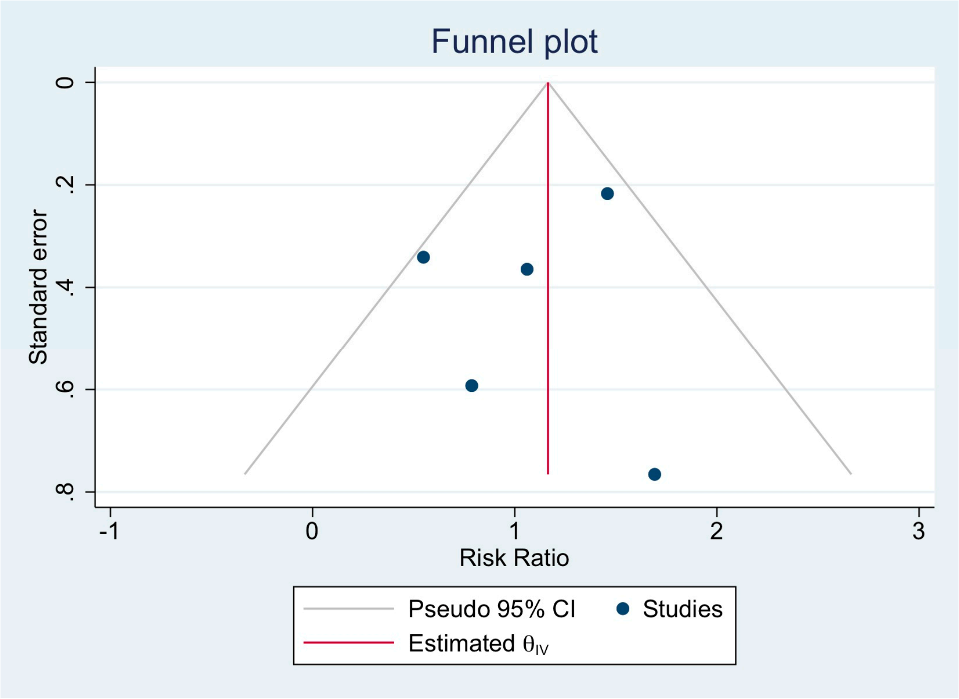

Supplementary Figure S14. Funnel plot of studies included in the morbidity analysis.

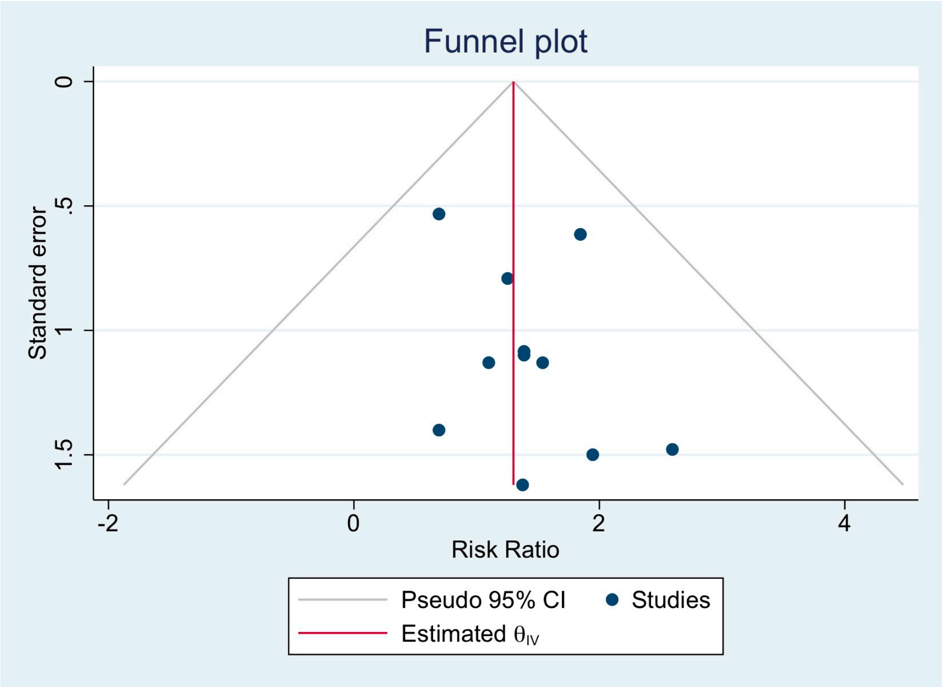

Supplementary Figure S15. Funnel plot of studies included in the ≥CDIII morbidity analysis.

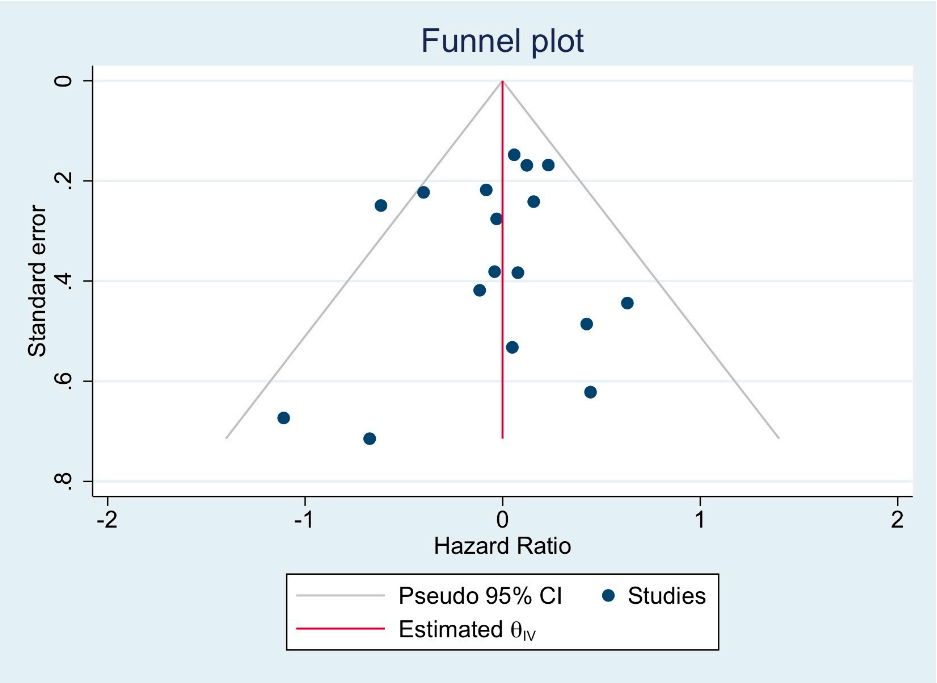

Supplementary Figure S16. Funnel plot of studies included in the mortality analysis.

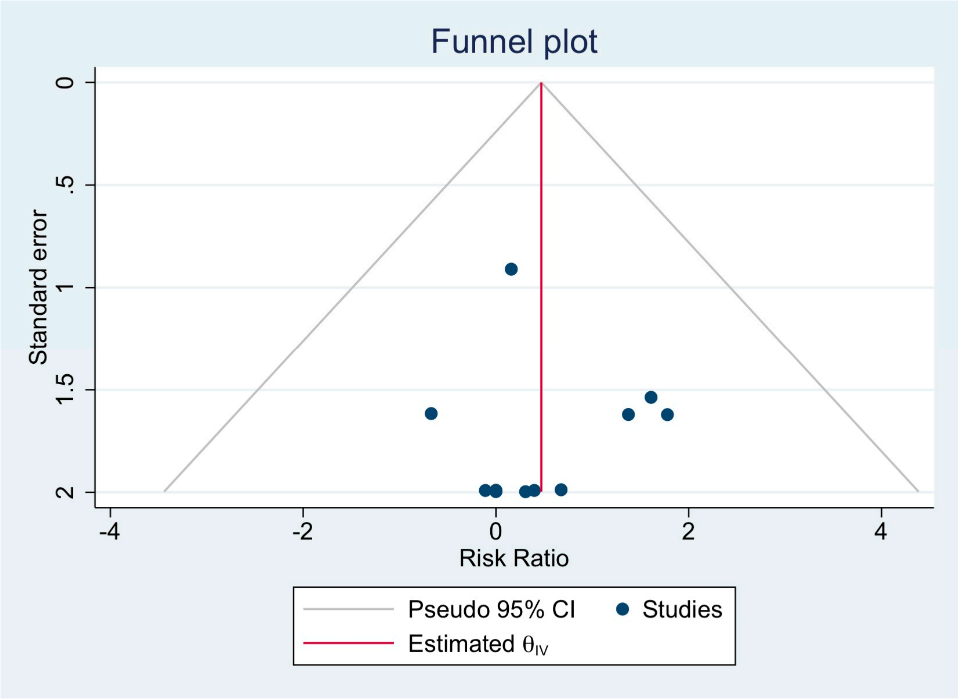

Supplementary Table S1. Risk of bias summary for non-randomized studies using the ROBINS-I tool. R1= Bias due to confounding, R2= Bias due to selection of participants, R3= Bias in classification of interventions, R4= Bias due to deviations of intended interventions, R5= Bias due to missing data, R6= Bias in measurement of outcomes, R7= Bias in selection of the reported results.

**Study**	**R1**	**R2**	**R3**	**R4**	**R5**	**R6**	**R7**	**Overall**
Chua	Low risk	Low risk	Low risk	Low Risk	Low risk	Low risk	Low risk	**Low risk of bias**
Zhong	Low risk	Low risk	Low risk	Low risk	Low risk	Low risk	Low risk	**Low risk of bias**
Wei	Low risk	Low risk	Low risk	Low risk	Moderate risk	Low risk	Low risk	**Low risk of bias**
Chen	Moderate Risk	Serious risk	Low risk	Low risk	Low Risk	Low risk	Low risk	**Serious risk of bias**
Matsumoto	Moderate risk	Low risk	Low risk	Low risk	Low Risk	Low risk	Low risk	**Low risk of bias**
Feng	Low risk	Low risk	Low risk	Low risk	Low risk	Low risk	Low risk	**Low risk of bias**
Saito	Serious risk	Low risk	Low risk	Low risk	Low risk	Low risk	Low risk	**Serious risk of bias**
Lu	Low risk	Low risk	Low risk	Low risk	Low risk	Low risk	Low risk	**Low risk of bias**
Yin	Moderate risk	Low risk	Low risk	Low risk	Serious risk	Low risk	Low risk	**Serious risk of bias**
Sun	Serious risk	Serious risk	Low risk	Low risk	Low Risk	Low risk	Moderate risk	**Serious risk of bias**
Wang	Moderate risk	Low risk	Low risk	Low risk	Moderate risk	Low risk	Low risk	**Moderate risk of bias**
Song	Low risk	Serious risk	Low risk	Moderate risk	Low risk	Low risk	Low risk	**Serious risk of bias**
Ho	Moderate risk	Low risk	Low risk	Low risk	Low risk	Low risk	Low risk	**Low risk of bias**
Chan	Moderate risk	Low risk	Low risk	Low risk	Low risk	Low risk	Low risk	**Moderate risk of bias**
Umeda	Moderate risk	Low risk	Low risk	Low risk	Low risk	Low risk	Low risk	**Moderate risk of bias**
Liang	Moderate risk	Low risk	Low risk	Moderate risk	Low risk	Low risk	Low risk	**Moderate risk of bias**

Supplementary Table S2. Risk of bias summary for randomized controlled trials using the RoB 2 tool. R1= Bias arising from the randomization process, R2= Bias due to deviations from intended intervention, R3= Bias due to missing outcome data, R4= Bias in measurement of the outcome, R5= Bias in selection of reported result.

**Study**	**R1**	**R2**	**R3**	**R4**	**R5**	**Overall**
Xia	Low risk	Low risk	Low risk	Low risk	Low risk	**Low risk of bias**
